# Propolis Exerts Antibiofilm Activity Against Methicillin-Resistant *Staphylococcus aureus* by Modulating Gene Expression to Suppress Adhesion

**DOI:** 10.3390/microorganisms13122810

**Published:** 2025-12-10

**Authors:** He Sang, Kaiyue Feng, Yanhu Ju, Yueying Sun, Yang Zhang, Hongzhuan Xuan, Fei Wang

**Affiliations:** 1 School of Pharmaceutical Sciences and Food Engineering, Liaocheng University, Liaocheng 252059, China; 2 College of Agriculture and Biology, Liaocheng University, Liaocheng 252059, China; 3 Shandong Key Laboratory of Applied Technology for Protein and Peptide Drugs, Liaocheng University, Liaocheng 252059, China; 4 School of Medicine, Liaocheng University, Liaocheng 252059, China

**Keywords:** propolis, methicillin-resistant *Staphylococcus aureus*, adhesion, biofilm

## Abstract

Within the global epidemiological landscape, methicillin-resistant *Staphylococcus aureus* (MRSA) stands out as a major contributor to infectious disease burden. The persistent public health crisis it presents arises from a dual challenge: intrinsic multidrug resistance coupled with a high rate of healthcare-associated infections. Recent studies have shown that propolis has unique advantages in bacterial infection prevention and treatment. The present study revealed that propolis ethanolic extract (PEE) exhibited notable antibacterial activity against both MRSA ATCC 43300 and MRSA CI2, with a minimum inhibitory concentration (MIC) of 128 μg/mL for each strain. Crystal violet (CV) staining and XTT sodium reduction assays were employed to evaluate the anti-biofilm efficacy of PEE. CV staining revealed that PEE significantly inhibited biofilm formation and reduced the biomass of pre-formed biofilm. Additionally, the XTT sodium reduction assay demonstrated a substantial reduction in the metabolic activity of the biofilm-embedded. Scanning electron microscopy and bacterial adhesion experiments revealed that PEE significantly reduced bacterial adhesion and aggregation. Furthermore, experiments on the synthesis of extracellular polysaccharides and proteins showed that PEE inhibits the production of water-soluble and alkali-soluble polysaccharides and extracellular proteins. Real-time quantitative Polymerase Chain Reaction (RT-qPCR) analysis revealed that PEE inhibited the expression of *icaADBC*, *fnbAB*, *clfAB*, and *sarA*. These results revealed that PEE inhibits biofilm formation and development by inhibiting the expression of *sarA*, *icaADBC*, *fnbAB*, and *clfAB*, thereby reducing the synthesis of extracellular polysaccharides and proteins to attenuate the adhesion capacity of MRSA. In summary, this study provides experimental evidence for the development of PEE as a potential antimicrobial agent for the prevention and treatment of MRSA-associated infections. Future work will focus on identifying its key active monomers and investigating its therapeutic effects and mechanisms of action in animal models.

## 1. Introduction

The irrational use of antibiotics and rising bacterial resistance have become global challenges. The diminishing returns of antibiotic therapy, a direct outcome of their overuse, now confront us with a critical worldwide health dilemma [1,2]. This reality is perhaps most concretely evidenced by the expanding reach of methicillin-resistant *Staphylococcus aureus* (MRSA) and demands decisive intervention. MRSA exhibits multidrug resistance via multiple mechanisms, including the expression of specialised drug-resistance and penicillin-binding proteins (PBP2a), which compensate for the transpeptidase function of PBP2 that is inhibited by antibiotics. Other mechanisms include the production of β-lactamase, which hydrolyses β-lactam antibiotics; reduces drug accumulation; and alters antibiotic targets and the expression of drug-modifying enzymes. The clinical threat of MRSA arises from its multidrug resistance. This includes not only methicillin but also other major classes (e.g., β-lactams, quinolones) and, critically, has evolved to compromise newer antibiotics like vancomycin and linezolid, rendering several last-line therapies ineffective [3]. This significantly limits the treatment options for life-threatening MRSA infections. Existing antibiotics are much less effective against MRSA, which greatly limits the clinical treatment options and poses significant challenges [2]. In India, the prevalence of MRSA infections has increased from 29% in 2009 to 47% in 2014 [4]. According to data from the China Bacterial Resistance Monitoring Network for 2023, the detection rate of MRSA was 29.6%, and the resistance rate to various antibiotics was high [5]. Infection and mortality rates among patients are rising, as is the isolation rate for nosocomial infections [6,7,8]. In a landmark effort to guide antimicrobial development, the World Health Organization designated methicillin-resistant Staphylococcus aureus (MRSA) as a high-priority pathogen in 2017 [9].

Expanding research on resistance mechanisms indicates that bacterial biofilm formation is a primary driver of MRSA resistance, which must be addressed to enhance treatment efficacy of *S. aureus* infections [10]. A biofilm represents an architecturally complex community of microorganisms embedded within a self-produced matrix of extracellular polymeric substances (EPS), which primarily include extracellular polysaccharides, proteins, and DNA (eDNA) [11]. The development of a biofilm is characterized by a dynamic progression through a series of morphological and functional changes, involving a sequence that is often described as comprising four key phases: (1) adhesion, (2) aggregation and proliferation, (3) maturation, (4) shedding and dissemination [12]. According to an estimate from the National Institutes of Health, around 65% of all MRSA infections are attributable to those involving bacterial biofilms [13]. Compared with planktonic bacteria, those in biofilm have slower metabolism and are insensitive to antibiotics. The development of a biofilm confers upon bacteria a significant survival advantage, leading to biofilm-mediated resistance that can enhance tolerance to antimicrobial agents by up to three orders of magnitude compared to free-living cells [14,15]. It is difficult for conventional antibiotics to penetrate biofilm, and they protect bacteria from host immune response, facilitating antibiotic removal. This enhances the pathogenicity of bacteria and restricts the effectiveness of antibiotics [16]. Consequently, biofilm formation confers upon MRSA significantly enhanced resistance to current clinical antibiotics, thereby constraining the therapeutic arsenal and greatly complicating treatment outcomes [17]. Given the severe challenges posed by MRSA biofilm, there is a critical imperative to develop innovative therapeutic strategies that are both safe and effective against biofilm-associated infections. The inherent structural complexity and distinctive bioactive profiles of natural products underpin their status as indispensable reservoirs for the development of novel antibacterial agents [18]. Propolis is a natural product primarily composed of flavonoids, phenolic acids, terpenes, and other compounds. These components have antibacterial, antioxidant, anti-inflammation, and antitumor effects, and are widely used for the management of various infectious diseases [19].

Bees produce propolis by collecting resins from plant buds and exudates, forming a chemically complex adhesive that serves as the colony’s primary natural defense. It is renowned for its diverse biological activities, largely due to its rich flavonoid, phenolic acid and ester content. These compounds are responsible for its well-documented antimicrobial, anti-inflammatory and antioxidant properties. The chemical profile and resultant bioactivity of propolis depend heavily on its botanical and geographical origins. Extensive evidence supports the potent antibacterial activity of propolis, validating its potential for further antimicrobial research [19,20,21]. However, studies on the activity of propolis against MRSA biofilms remain limited, and research on its inhibitory mechanism toward MRSA biofilm is still in its preliminary stages. Therefore, the objective of this work was to assess the anti-biofilm activity of poplar propolis ethanolic extract against both reference (ATCC 43300) and clinical (CI2) MRSA strains, and to explore its potential mode of action. These results establish a compelling rationale for exploring poplar propolis in future strategies to combat MRSA infections.

## 2. Materials and Methods

### 2.1. Propolis Substances and Bacterial Strains

The propolis used in this study was sourced from Shandong Province, China, during 2022. The collected propolis is characterized as being primarily derived from poplar (*Populus* sp.) exudates. MRSA ATCC 43300 and the clinical isolate MRSA CI2 were sourced from and maintained at the Natural Product Bioactivity Laboratory, School of Pharmaceutical Sciences and Food Engineering, Liaocheng University.

### 2.2. Preparation of Propolis Ethanol Extract

Raw poplar propolis was ground into a powder. The powder was extracted by maceration in 95% ethanol (1:10, *w*/*v*) with continuous shaking (150 rpm) at 37 °C for 48 h. After initial filtration using a Whatman No.4 filter (Cytiva, Shanghai, China), the extract was refrigerated (4 °C, 24 h) to dewax. The chilled mixture was clarified by passage through a Whatman No.1 filter (Cytiva, Shanghai, China). The resulting clarified filtrate was concentrated under reduced pressure using a rotary evaporator (YRE-2000B, Yuhua, Gongyi, China) to obtain the propolis ethanolic extract (PEE). The obtained PEE paste was stored at −20 °C until use. All experimental solutions were prepared fresh immediately prior to each assay.

### 2.3. UPLC-MS/MS Analysis

UPLC-MS/MS analysis (Sciex 4500, Sciex, Boston, MA, USA) was employed for the chemical profiling of PEE.

A HYPERSIL GOLD C18 column (100 mm × 2.1 mm, 3 μm; 35 °C) was used for separation. The mobile phase comprised (A) 0.1% formic acid in ultrapure water and (B) acetonitrile, with the flow rate maintained at 0.3 mL/min. A gradient elution program was applied: 0–2 min, 90% A; 2–6 min, 90% A to 10% A; 6–8 min, 10% A; 8–8.1 min, 10% A to 90% A; 8.1–10 min, 90% A (re-equilibration). The injection volume was set at 1 μL.

Mass spectrometric analysis was conducted using an electrospray ionization (ESI) source operating in positive/negative switching mode. Key source parameters were configured as follows: ion spray voltage at 5500 V (positive) and −4500 V (negative), source temperature at 550 °C, with curtain gas and collision gas (N_2_) pressures maintained at 30 psi and 9 psi, respectively. Data were acquired in multiple reaction monitoring (MRM) mode. For each target compound, optimal precursor-product ion transitions, along with their corresponding declustering potentials (DPs) and collision energies (CEs), were determined using authentic standards. Compounds were identified by matching both retention times and MRM transitions with those of the reference standards (Solarbio, HPLC ≥ 98%, Beijing, China). Quantification was performed based on integrated chromatographic peak areas using an external standard calibration curve.

### 2.4. Determination of the Minimum Inhibitory Concentration (MIC)

Using the broth microdilution method, we determined the MIC of PEE against two MRSA strains, ATCC 43300 and CI2 [22]. The bacterial strains MRSA ATCC 43300 and MRSA CI2 were cultured at 37 °C to obtain bacterial suspension. The bacterial suspension was adjusted with trypsin soybean broth (TSB) to a final concentration of 3 × 10^5^ CFU/mL. Then, 100 μL of PEE at different concentrations was mixed with 100 μL of bacterial suspension at a ratio of 1:1 in a 96-well plate. The final concentration gradient of PEE in the mixture was set to 8 to 256 μg/mL, with incubation at 37 °C for 24 h. A 20 μL volume of 1 mg/mL resazurin sodium solution (Psaitong, ≥80%, Jinming, Beijing, China) was added to all wells to assess metabolic activity. The plates underwent a 3 h, 37 °C incubation in the dark prior to visual inspection of the color reaction. TSB and vancomycin hydrochloride (50 μg/mL) were used as negative (control) and positive controls, respectively. The minimum PEE concentration required to prevent the color of the solution from changing from purple to orange was determined as the MIC.

### 2.5. Determination of the Minimum Bactericidal Concentration (MBC)

Transfer 50 µL of the bacterial suspension from the wells with PEE concentrations equal to or greater than the MIC. Samples were evenly spread on TSB agar plates, followed by incubation at 37 °C for 24 h, and the resulting colonies were counted. The MBC is defined as the lowest PEE concentration that results in no visible colonies on the TSB agar plate after subculturing.

### 2.6. Anti-Biofilm Assay

#### 2.6.1. Inhibitory Effect of PEE on MRSA Biofilm Formation

Two strains of MRSA were diluted to 3 × 10^7^ CFU/mL in TSB medium. Subsequently, in 96-well plates, the prepared bacterial suspension was exposed to PEE, so that the final concentrations of PEE were 128, 64, 32, 16, and 8 μg/mL, respectively. Then, bacterial inoculation into 1% (*w*/*v*) sucrose-supplemented TSB in 96-well plates was followed by a 24 h incubation at 37 °C. To remove non-adherent planktonic cells, the supernatant was carefully aspirated, and the wells were subjected to three gentle washes with phosphate-buffered saline (PBS, pH 7.4). The total amount and activity of MRSA biofilm were measured using crystal violet (CV, Macklin, AR, Shanghai, China) staining and XTT sodium (Psaitong, 90%, Jinming, Beijing, China) reduction assays, respectively. The optical density (OD) values at 590 nm and 490 nm were read on a microplate reader (FlexA-200, Aosheng, Hangzhou, China).

#### 2.6.2. Inhibitory Effect of PEE on Mature MRSA Biofilm

MRSA suspension (1.5 × 10^7^ CFU/mL) was added to 96-well plate, with each well subsequently supplemented with 1% sucrose. To allow for biofilm formation, the plates were incubated at 37 °C for 24 h. Following incubation, the supernatant containing non-adherent bacteria was aspirated, and the wells were gently washed three times with PBS. PEE (8–128 μg/mL) was added and the control group was established. The plates were then returned to the 37 °C incubator for an additional 24 h. After this second incubation, the treatment supernatant was discarded, and the adherent biofilm was washed three times with PBS. Finally, CV staining and XTT sodium reduction assays conducted used to evaluate the biomass and cell activity of the MRSA biofilm.

#### 2.6.3. Scanning Electron Microscopy (SEM) of Biofilm

SEM was performed to further analyze the effect of PEE on the microstructure of MRSA biofilm. For biofilm formation, sterile coverslips were positioned within the wells of a six-well plate, which were then inoculated with an MRSA suspension (3 × 10^7^ CFU/mL in 1% sucrose) and subsequently incubated at 37 °C for 24 h. After this period, the planktonic culture was aspirated, and the biofilms were treated with the respective PEE concentrations (control, 64 μg/mL), followed by an additional 24 h incubation. After incubation, they were rinsed with buffer and fixed with 2.5% glutaraldehyde solution (Macklin, AR,50% in H_2_O, Shanghai, China) overnight at 4 °C. After dehydration using an ethanol gradient and treatment with tert-butanol (Macklin, AR, ≥99.0%, Shanghai, China), the fixed samples were freeze-dried. Finally, the dried samples were coated with gold and the microstructures of the biofilm were observed using SEM (S-4800, Hitachi, Tokyo, Japan).

#### 2.6.4. Determination of Bacterial Adhesion

MRSA suspension (100 μL, 3 × 10^8^ CFU/mL) was added to each well of the 96-well plate, followed by an equal volume of TSB medium (containing PEE and 1% sucrose). The final concentration of PEE was set at 8–256 μg/mL, and the control group was TSB containing 1% sucrose. Incubation was carried out at 37 °C across specified time points: 2, 4, 6, 8, 10, and 12 h. At each time point, the supernatant was aspirated, and adherent cells were washed three times with PBS. Subsequently, 200 μL of TSB medium was added to each well. After ultrasonication for 5 min, for quantification of bacterial adhesion, the OD at 600 nm was recorded using a FlexA-200 microplate reader (Aosheng, Hangzhou, China).

#### 2.6.5. Analysis of Extracellular Polysaccharide Production

For quantification of extracellular polysaccharides by the phenol/sulfuric acid method, MRSA cells (3 × 10^8^ CFU/mL) were subjected to PEE treatment (8, 16, 32 and 64 μg/mL) at 37 °C for 24 h. MRSA were harvested by centrifugation (12,000× *g*, 30 min, 4 °C). The precipitate was then washed by resuspension in sterile water. The suspension was then centrifuged again (12,000× *g*, 4 °C, 30 min), and the supernatant containing water-soluble polysaccharide was collected. The pellet was resuspended in 0.1 mol/L NaOH and then subjected to centrifugation (12,000× *g*, 4 °C, 30 min). This process of collecting the supernatant and recentrifuging was repeated three times. The combined supernatants were then mixed with three volumes of 95% ethanol and stored at 4 °C overnight to precipitate the alkali-soluble polysaccharides, which were recovered by final centrifugation.

#### 2.6.6. Analysis of Extracellular Proteins Synthesis

To evaluate the effect of PEE on the secretion of extracellular proteins by MRSA, the bacteria were cultured in TSB medium until they reached a concentration of approximately 3 × 10^8^ CFU/mL. The bacterial suspension was then mixed with an equal volume of PEE solution at various sub-inhibitory concentrations (final concentrations of 8, 16, 32 and 64 μg/mL). The mixture was incubated at 37 °C for 24 h. The TSB medium was removed by low-speed centrifugation. Then, the precipitate was then resuspended using PBS pipetting and shaking, after which the bacterial cells were removed by centrifugation. The clarified supernatant was aspirated, ensuring it was free from residual cells or debris. The extracellular protein content of the supernatant was quantified using a Bradford protein detection kit (Jiancheng, Nanjing, China).

#### 2.6.7. Real-Time Quantitative Polymerase Chain Reaction (RT-qPCR) Analysis of Gene Expression

MRSA expresses extracellular polysaccharides and proteins that enable the bacteria to adhere, thereby promoting biofilm formation and aggregation. RT-qPCR was used to evaluate the effect of PEE on the expression of associated genes.

The pre-cultured MRSA bacterial suspension was incubated in TSB medium containing 1% sucrose and PEE (8–32 μg/mL) during a 12 h incubation at 37 °C. Following incubation, cells were harvested by centrifugation (12,000× *g*, 4 °C, 30 min). Total RNA was then isolated from the pellet using the Flying Shark Plus Bacteria RNA Kit (Nobelab, Jinan, China). According to the manufacturer’s protocol, the purified RNA was reverse-transcribed into complementary DNA (cDNA) employing the ReScript IV RT SuperMix for qPCR (Nobelab). The cDNA was used as a template for RT-qPCR using 2 × SYBR Premix UrTaqT II (Nobelab) on a QuantStudio Applied Biosystems system (Applied Biosystems, Waltham, MA, USA). Notably, normalization was performed against 16S cDNA, and relative gene expression was calculated using the 2(−ΔΔCT) method from the obtained CT values. Table 1 details the primer sequences for RT-qPCR amplification of the target genes.

### 2.7. Statistical Analysis

All experiments were repeated independently at least three times, and the data were expressed as the mean ± SD. Analysis of variance (ANOVA) followed by Tukey’s multiple comparisons test were performed using GraphPad Prism 9.5.0 software. Data were considered statistically significant at * *p* < 0.05.

## 3. Results

### 3.1. PPE Chemical Composition

The major compounds identified in PEE are shown in Table 2. The ten quantitatively analysed compounds include the following flavonoids: naringenin, apigenin, chrysin, kaempferol, galangin, and quercetin; phenolic acids: ferulic acid and caffeic acid; organic acids: cinnamic acid; and phenolic acid esters: phenethyl caffeate. The antibacterial properties of most of the identified compounds, particularly the abundant ones listed in Table 2, are well documented in the literature [27,28,29,30,31,32,33]. The variety of chemical components and combination of active ingredients in propolis provide it with additional antimicrobial agent [34].

### 3.2. Antibacterial Activity

The in vitro antibacterial activity of PEE against MRSA was evaluated using the MIC and MBC assays. PEE demonstrated potent antibacterial activity against both MRSA ATCC 43300 and MRSA CI2 strains. The corresponding MIC and MBC values are presented in Table 3.

### 3.3. Effect of PEE on Biofilm Formation

**Figure 1:**
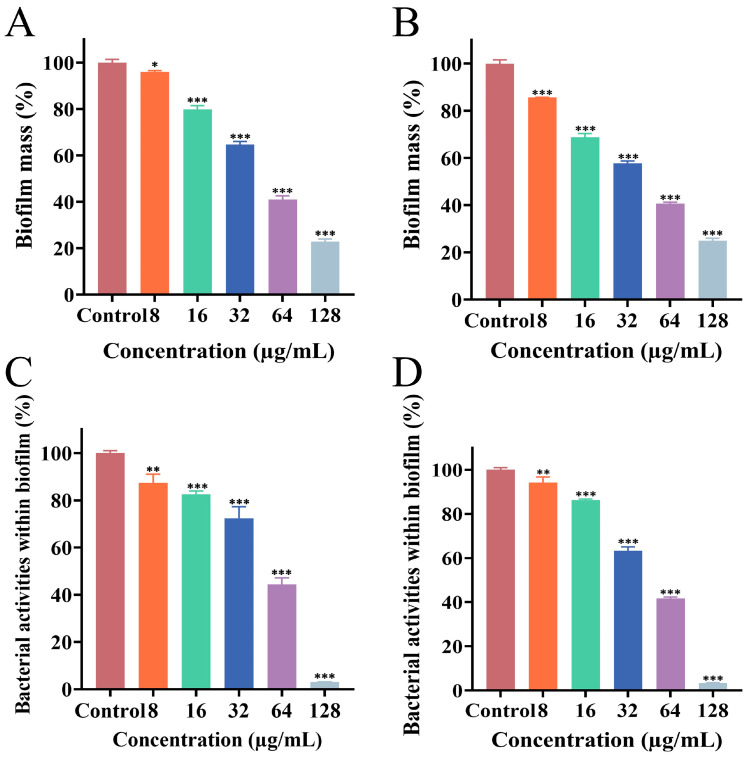
Effects of different concentrations of PEE on MRSA ATCC 43300 biofilm formation (**A**,**C**) and MRSA CI2 biofilm formation (**B**,**D**). (**A**,**B**) Biofilm biomass amount assessed by CV staining assay; (**C**,**D**) biofilm activity assessed by XTT sodium reduction assay (* *p* < 0.05, ** *p* < 0.01, and *** *p* < 0.001).

To explore the effect of PEE on biofilm formation by the MRSA ATCC 43300 and CI2 strains, biofilm mass and activity were evaluated using quantitative analysis. The experimental results showed that (Figure 1A–D), PEE treatment resulted in a concentration-dependent decrease in both biofilm biomass and metabolic activity for the two MRSA strains, compared with the untreated control group. Biofilm formation was significantly inhibited at a PEE concentration of 8 μg/mL, with this effect becoming more pronounced as the concentration increased. Taken together, the data indicate that PEE exerts a notable inhibitory effect on nascent biofilm formation.

### 3.4. Effect of PEE on Mature Biofilm

**Figure 2:**
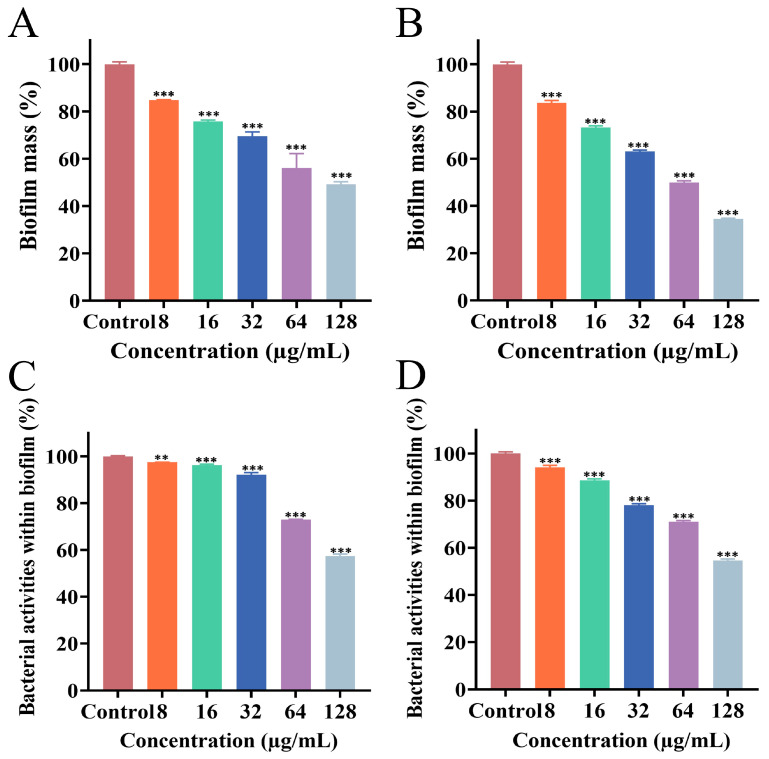
Effects of different concentrations of PEE on mature MRSA ATCC 43300 biofilm (**A**,**C**) and mature MRSA CI2 biofilm (**B**,**D**). (**A**,**B**) Biofilm biomass assessed by CV staining assay; (**C**,**D**) biofilm activity amount assessed by XTT sodium reduction assay (** *p* < 0.01, *** *p* < 0.001).

The effects of PEE on mature biofilm of MRSA ATCC 43300 and MRSA CI2 strains were detected using CV staining and the XTT sodium reduction assay to assess biofilm mass and activity, respectively. Figure 2A–D depicts the corresponding dose–response effects.

Compared with the control group, the treatment group exhibited a significant inhibitory effect on both the mass and activity of biofilm formed by both bacterial strains. When the concentration of PEE reached 8 μg/mL, both the mass and activity of MRSA biofilm were markedly reduced, with the extent of inhibition increasing as the concentration of PEE increased. The results showed that PEE exerts a substantial inhibitory effect on the mature biofilm of both strains.

### 3.5. Effect of PEE on Biofilm Structure

**Figure 3:**
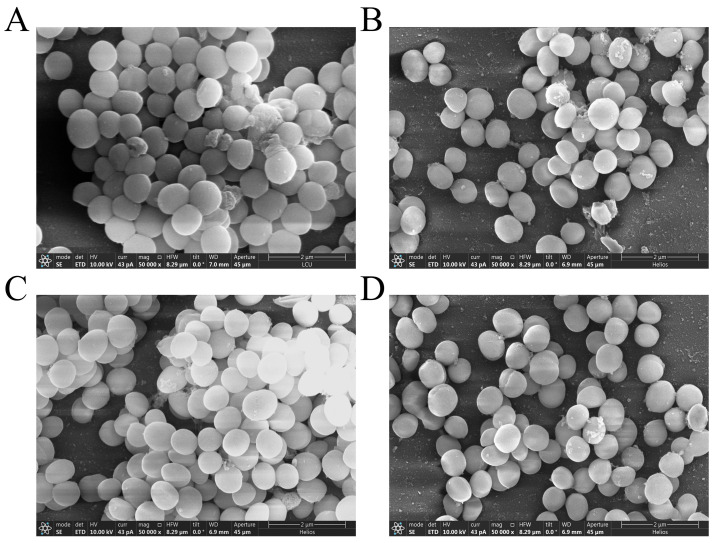
SEM photos of MRSA ATCC 43300 and MRSA CI2 biofilm treated with different concentrations of PEE. (**A**) SEM photo of MRSA ATCC 43300 biofilm without PEE treatment. (**B**) SEM photo of MRSA ATCC 43300 biofilm treated with 64 μg/mL of PEE. (**C**) SEM photo of MRSA CI2 biofilm without PEE treatment. (**D**) SEM photo of MRSA CI2 biofilm treated with 64 μg/mL of PEE.

SEM was used to assess the effect of PEE on MRSA biofilm formation on coverslips (Figure 3). In the absence of PEE, MRSA was uniformly distributed on the coverslips, the cells were tightly arranged and formed a dense biofilm, and the bacterial cells adhered to each other. However, when MRSA was co-cultured with 64 μg/mL of PEE, biofilm formation was reduced. The biofilm cells loosened, and the thickness and connections between the cells were reduced. The adhesion and aggregation were also significantly reduced. These results indicate that PEE inhibits the adhesion and aggregation of MRSA and reduces the formation and consolidation of biofilm.

### 3.6. Effect of PEE on Bacterial Adhesion

**Figure 4:**
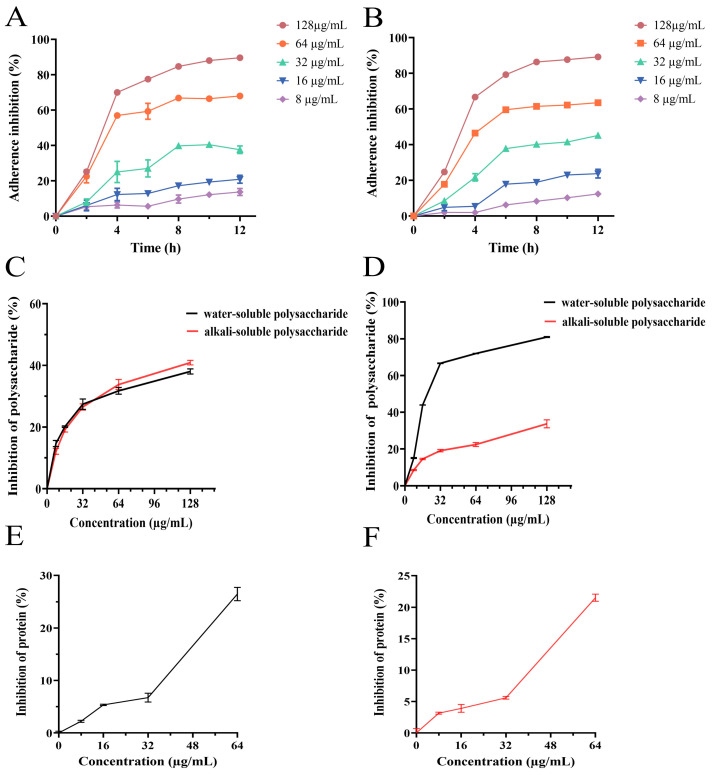
Effect of different concentrations of PEE on MRSA bacterial adhesion, extracellular polysaccharide generation and extracellular protein formation. (**A**) PEE suppresses adhesion of MRSA ATCC 43300; (**B**) PEE suppresses adhesion of MRSA CI2. (**C**) PEE inhibited the production of water-soluble polysaccharide and alkali-soluble polysaccharide of MRSA ATCC 43300; (**D**) PEE inhibited the production of water-soluble polysaccharide and alkali-soluble polysaccharide of MRSA CI2. (**E**) Extracellular protein content of MRSA ATCC 43300; (**F**) Extracellular protein content of MRSA CI2.

Biofilm development constitutes a dynamic, multi-stage process, in which bacterial adhesion serves as the critical initial step for subsequent establishment and maturation [35]. In this study, the effect of propolis on MRSA adhesion was evaluated using in vitro adhesion experiments. As shown in Figure 4A,B, the bacterial adhesion rate in the propolis treatment group was significantly lower than that in the control group. In addition, the inhibition rate was related to the culture time. PEE not only inhibited bacterial growth but also affected their ability to adhere to solid surfaces. Therefore, the ability of PEE to reduce bacterial adhesion may be one of its mechanisms of action against MRSA biofilm.

### 3.7. Effect of PEE on Extracellular Polysaccharides Synthesis

Extracellular polysaccharides play a key role in bacterial adhesion. Alkali-soluble polysaccharides are extracellular glucans that play important roles in biofilm formation and consolidation. Water-soluble polysaccharides provide nutrients for adherent and aggregated bacteria. In this study, a phenol–sulfuric acid assay was performed to determine the effect of PEE on the synthesis of MRSA alkali- and water-soluble polysaccharides. The results are shown in Figure 4C,D. Relative to untreated control, after treatment with PEE at a concentration of 8–64 μg/mL, PEE significantly inhibited the synthesis of extracellular polysaccharides in a significant concentration-dependent manner. These findings demonstrate that PEE significantly inhibited the synthesis of extracellular polysaccharides by MRSA.

### 3.8. Effect of PEE on Extracellular Proteins Synthesis

MRSA plays a significant role in bacterial adhesion, as shown in Figure 4E,F. Treatment with PEE (8–64 μg/mL) significantly decreased protein content in a dose-dependent manner. This indicates that a decrease in the protein content was positively correlated with the PEE concentration. These findings suggest that PEE inhibited biofilm formation and consolidation by reducing extracellular protein production.

### 3.9. Effect of PEE on Biofilm-Related Gene Expression

**Figure 5:**
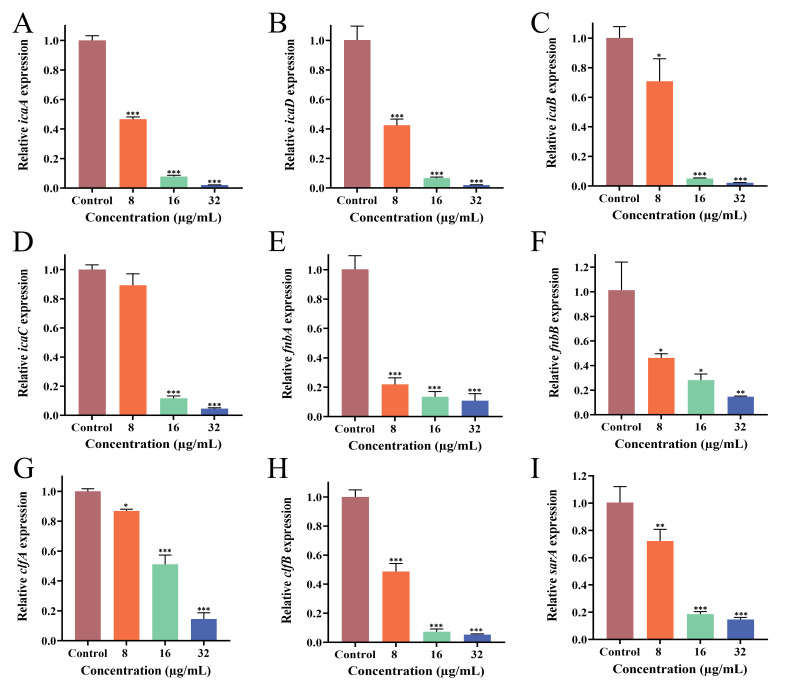
Effects of different concentrations of PEE on the expression of the *icaA*, *icaD*, *icaB, icaC, fnbA*, *fnbB, clfA, clfB* and *sarA* genes of MRSA ATCC 43300. (**A**) PEE inhibits the expression of *icaA*; (**B**) *icaD*; (**C**) *icaB*; (**D**) *icaC*; (**E**) *fnbA*; (**F**) *fnbB*; (**G**) *clfA*; (**H**) *clfB*; (**I**) *sarA*. (* *p* < 0.05, ** *p* < 0.01, and *** *p* < 0.001).

**Figure 6:**
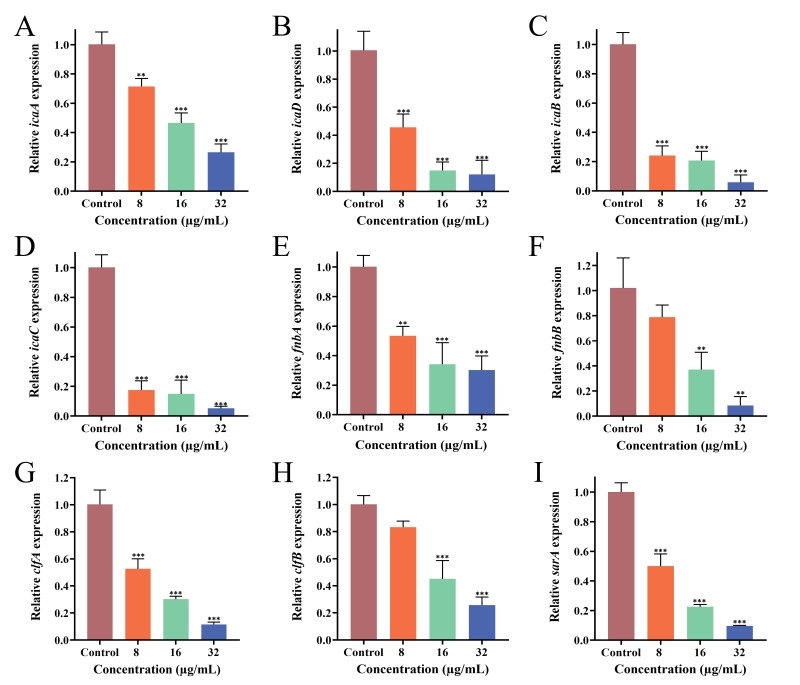
Effects of different concentrations of PEE on the expression of the *icaA*, *icaD*, *icaB, icaC, fnbA*, *fnbB, clfA, clfB* and *sarA* genes of MRSA CI2. (**A**) PEE inhibits the expression of *icaA*; (**B**) *icaD*; (**C**) *icaB*; (**D**) *icaC*; (**E**) *fnbA*; (**F**) *fnbB*; (**G**) *clfA*; (**H**) *clfB*; (**I**) *sarA*. (** *p* < 0.01, and *** *p* < 0.001).

To elucidate the mechanism by which PEE inhibited MRSA biofilm formation and consolidation, we examined the relative expression of biofilm-associated genes. Extracellular polysaccharides and proteins are also closely associated with bacterial adhesion. The main component of the extracellular polysaccharides is polysaccharide intercellular adhesin (PIA), which is regulated by the *icaADBC* gene. Fibronectin-binding proteins (FnbpA and FnbpB) and aggregation factors (ClfA and ClfB) are the extracellular proteins regulated by *fnbAB* and *clfAB*, respectively. The *sarA* gene is an important regulatory gene in *S. aureus* and is primarily responsible for the regulation of biofilm formation and consolidation. The expressions of *icaADBC*, *fnbAB*, *clfAB*, and *sarA* were significantly downregulated in MRSA ATCC 43300 (Figure 5) and MRSA CI2 (Figure 6). Compared with the control group, gene expression in the PEE treatment group was significantly inhibited, and the inhibitory effect became more obvious as the PEE concentration increased.

## 4. Discussion

MRSA is a Gram-positive bacterium whose infections are difficult to treat and carry a high mortality rate. Since its discovery, the isolation rate has been increasing annually and has become a serious clinical, medical, and public health problem [36,37]. Studies have shown that MRSA forms biofilm, making it more resistant to antibiotics and other defense mechanisms [9]. In addition, this makes the clinical treatment of MRSA infections increasingly difficult. Consequently, this persistent challenge necessitates the development of novel therapeutic strategies. As a natural component, propolis contains various physiologically active substances, such as flavonoids, terpenes, and organic acids [38].

In this study, the primary bioactive constituents of PEE of Shandong poplar propolis were quantitatively determined using UPLC-MS/MS analysis. The results demonstrate that chrysin, galangin, and phenethyl caffeate are the predominant compounds in PEE, a profile that is highly consistent with the established chemical fingerprint of poplar propolis in northern China [39]. This compositional pattern is of notable biological significance, as chrysin, galangin, and phenethyl caffeate have been shown to effectively disrupt bacterial biofilm [40,41]. Therefore, it is hypothesized that the overall biological activity of PEE is not attributable to any trace constituent, but rather results from the synergistic effects of multiple high-abundance bioactive components. This intricate composition establishes a rational foundation for its multi-target and multi-mechanistic mode of action, thereby explaining the occasionally superior efficacy observed in natural extracts relative to isolated pure compounds.

There has been growing scientific interest in the antibacterial properties of propolis in recent years, owing to its potential as a natural alternative. da Silva et al. (2025) reported that Brazilian green and brown propolis exhibited potent antibacterial activity against human dental pulp fibroblasts [42]. Wang et al. (2022) showed that Chinese propolis ethanol extract exhibited significant antibacterial effects against MRSA [43]. Based on a study conducted by Manginstar et al. (2025), stingless bee propolis has a dual function in modulating inflammation induced by MRSA and *Pseudomonas aeruginosa*, as well as in preventing bacterial infections in a second-degree burn model [44]. Studies have also shown that propolis sourced from mining regions in Romania possesses antibacterial activity against five bacterial strains (*S. aureus*, *Escherichia coli*, *Pseudomonas aeruginosa*, *Enterococcus faecalis*, and *Streptococcus mutans*) and five fungal strains (*Candida albicans*, *Aspergillus niger*, *Aspergillus flavus*, *Cryptococcus neoformans*, and *Penicillium chrysogenum*) [45]. In this study, we found that PEE collected from Shandong, China, exhibited significant antibacterial and anti-biofilm activities against MRSA. Its MIC value is at the same order of magnitude as that of other Chinese propolis activities reported by Feng et al., confirming its potential as an antibacterial lead compound [46]. Our results demonstrate that at sub-inhibitory concentrations, PEE can not only effectively inhibit the initial adhesion of biofilm, but also significantly reduce the biomass of mature biofilm.

Biofilm formation is a complex and multistage process. Bacterial adhesion constitutes a pivotal initial event, driving the subsequent formation and development of biofilm [47]. The main component of biofilm is EPS, which comprises three major components: extracellular polysaccharides, extracellular proteins, and eDNA. These components play a synergistic role in the formation and consolidation of biofilm and maintain their structural stability and biological functions [48]. In MRSA, the main extracellular polysaccharide component is PIA, which plays a key role in MRSA adhesion and aggregation. Extracellular proteins including fibronectin-binding proteins (FnbpA and FnbpB), aggregation factors (ClfA and ClfB), collagen-binding proteins (Cna), fibrinogen-binding proteins (Fib), laminin-binding proteins (Eno), and elastin-binding proteins (Ebps), are also involved in this crucial process. These proteins are collectively referred to as the microbial surface components that recognize adhesive matrix molecules [49,50,51]. They mediate specific adhesion between bacteria and surfaces or between bacteria by recognizing the extracellular matrix components of host cells. Our research found that PEE can significantly inhibit the formation and development of MRSA biofilm while reducing the contents of extracellular polysaccharides and proteins. Based on these experimental results, we speculate that the mechanism of action of PEE may involve two aspects: on one hand, it significantly downregulates PIA synthesis, and on the other hand, it may inhibit the generation of various extracellular proteins. This dual inhibitory effect eventually leads to the weakening of the adhesion and aggregation abilities of MRSA, providing a potential target for the development of new anti-biofilm drugs.

Multiple genes regulate biofilm formation and consolidation. In MRSA, the synthesis of PIA is mainly controlled by the *ica* operon, which consists of four functional genes, *icaA*, *icaD*, *icaB*, and *icaC*, which play synergistic but distinct roles in PIA synthesis [33]. *IcaA* encodes N-acetylglucosamine transferase and is a key catalytic enzyme involved in PIA synthesis. As a cofactor of *icaA*, *icaD* can significantly enhance the catalytic activity of *icaA* and improve the efficiency of PIA synthesis. *IcaB* encodes PIA deacetylase and endows it with a positive charge through the deacetylation of polysaccharide chains, promoting electrostatic stability and the structural integration of biological membrane matrices. *IcaC* encodes an outer membrane transporter that is responsible for effectively secreting extracellular synthetic PIA to form EPS [52]. In addition, the expression of microbial surface adhesion factors is regulated by genes such as *fnbAB*, *clfAB*, *can*, *fib*, *eno*, and *ebps*, among which *fnbAB* and *clfAB* function in the process of bacterial adhesion and aggregation. Notably, the *sarA* gene, a global regulatory factor, encodes the SarA protein and has positive regulatory effects on genes such as *icaADBC*, *fnbA* [33,53,54]. In the present study, PEE significantly inhibited *sarA*, *icaADBC*, *fnbpAB,* and *clfAB* expressions. Based on these findings, we hypothesize that the mechanism through which PEE acts against MRSA biofilm may involve downregulating *sarA*, *icaADBC*, *fnbpAB*, and *clfAB* gene expressions, leading to a reduction in the extracellular polysaccharide and protein contents of MRSA. This weakens the bacterial adhesion ability and ultimately inhibits biofilm formation and development. These findings clarify the mechanism by which PEE exerts antibiofilm activity and paves the way for novel strategies to combat biofilm-related infections.

In summary, we have constructed a model of the effect of PEE on MRSA biofilm: PEE, through its complex multi-component system, inhibits the expression of virulence factors at multiple targets at the transcriptional level and interferes with the formation and maintenance of biofilm at multiple stages at the phenotypic level. Future research will focus on isolating the main monomer compounds in PEE, verifying their synergistic interactions, and further revealing their upstream targets by using transcriptomics and other techniques, thereby providing a more solid basis for developing new strategies based on natural products to resist drug-resistant bacteria.

## 5. Conclusions

In summary, PEE exhibits potent anti-MRSA activity. Its anti-biofilm activity is significant and concentration-dependent, reducing both activity and mass. The significant anti-biofilm activity of PEE is manifested as a decrease in biofilm activity and biofilm mass, and it is concentration-dependent. The antibiofilm mechanism of PEE against MRSA was elucidated by analyzing bacterial adhesion, the production of extracellular polysaccharides, generation of extracellular proteins, and expression of related regulatory genes, which can inhibit the expression of *sarA*, *icaADBC*, *fnbAB*, and *clfAB*, thereby reducing the synthesis of extracellular polysaccharides and proteins, lowering bacterial adhesion ability, and achieving an antibiofilm effect. Therefore, PEE, as a natural drug candidate, shows broad developmental space. This study establishes a robust foundation for advancing its subsequent translational development, with a key focus on validating its efficacy in vivo. We intend to employ a clinically relevant infection model to systematically assess the clearance efficacy of PEE against MRSA in vivo, as well as its capacity to promote tissue repair. This well-defined research trajectory is critical for bridging the gap between existing in vitro findings and potential clinical applications and will provide essential evidence for elucidating the mechanism of action of PEE in complex physiological contexts.

## Figures and Tables

**Table 1:** Sequences of the primers in this study.

Gene	Primer Sequence (5′→3′)	Reference
*icaA*	F: CTTGCTGGCGCAGTCAATAC	[23]
	R: GTAGCCAACGTCGACAACTG	
*icaD*	F: TGGGCATTTTCGCGATTATCA	[23]
	R: ACGATTCTCTTCCTTTCTGCCA	
*icaB*	F: CCTGTAAGCACACTGGATGG	[23]
	R: TCGCTTTTCTTACACGGTGA	
*icaC*	F: TGCGTTAGCAAATGGAGACT	[23]
	R: TGCGTGCAAATACCCAAGAT	
*fnbA*	F: AAATTGGGAGCAGCATCAGT	[24]
	R: GCAGCTGAATTCCCATTTTC	
*fnbB*	F: CAACCAGTCGTTAAGCTCTGTGAC	[25]
	R: GCTGACATCATCAAGCTTTGC	
*clfA*	F: ACCCAGGTTCAGATTCTGGCAGCG	[24]
	R: TCGCTGAGTCGGAATCGCTTGCT	
*clfB*	F: ACATCAGTAATAGTAGGGGGCAAC	[26]
	R: TTCGCACTGTTTGTGTTTGCAC	
*sarA*	F: GTAATGAGCATGATGAAAGAACTGT	[23]
	R: CGTTGTTTGCTTCAGTGATTCG	
16S rRNA	F: CGCAATGGGCGAAAGC	[23]
	R: TACGATCCGAAGACCTTCATCA	

**Table 2:** Major compounds identified from PEE by UPLC-MS/MS.

Compounds	Structural Formula	Molecular Formula	Retention Time (min)	Content (mg/g)
caffeic acid	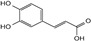	C_9_H_8_O_4_	3.47	3.93 ± 0.068
ferulic acid	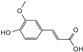	C_10_H_10_O_4_	5.13	0.78 ± 0.001
Quercetin	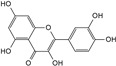	C_15_H_10_O_7_	5.72	1.01 ± 0.027
Apigenin	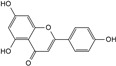	C_15_H_10_O_5_	5.94	0.73 ± 0.006
cinnamic acid	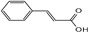	C_9_H_8_O_2_	5.96	0.11 ± 0.005
kaempferol	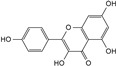	C_15_H_10_O_6_	5.98	0.66 ± 0.007
Naringenin	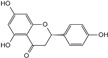	C_15_H_12_O_5_	6.05	1.41 ± 0.029
Chrysin	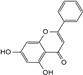	C_15_H_10_O_4_	6.55	12.38 ± 0.426
Galangin	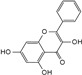	C_15_H_10_O_5_	6.61	8.37 ± 0.042
phenethyl caffeate	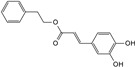	C_17_H_16_O_4_	6.65	8.84 ± 0.150

**Table 3:** The minimum inhibitory concentration (MIC) and the minimum bactericidal concentration (MBC) values of PEE against MRSA ATCC 43300 and MRSA CI2.

Compound	Strain	MIC (µg/mL)	MBC (µg/mL)
PEE	MRSA ATCC 43300	128	256
	MRSA CI2	128	256

## Data Availability

The original contributions presented in this study are included in the article. Further inquiries can be directed to the corresponding author.

## Abbreviations

The following abbreviations are used in this manuscript:

MRSA	Methicillin-Resistant *Staphylococcus aureus*
PEE	Propolis Ethanolic Extract
MIC	Minimum Inhibitory Concentration
XTT	2,3-Bis-(2-methoxy-4-nitro-5-sulfophenyl)-2H-tetrazolium-5-carboxanilide
CV	Crystal Violet
SEM	Scanning Electron Microscopy
PBP2a	Penicillin-Binding Proteins 2a
CFU	Colony-Forming Units
TSB	Trypticase soy broth
PBS	Phosphate-Buffered Saline
OD	Optical Density
RT-qPCR	Real-time quantitative Polymerase Chain Reaction
PIA	Polysaccharide Intercellular Adhesin
